# Management of thyroid cancer: United Kingdom National Multidisciplinary Guidelines

**DOI:** 10.1017/S0022215116000578

**Published:** 2016-05

**Authors:** A L Mitchell, A Gandhi, D Scott-Coombes, P Perros

**Affiliations:** 1 The Newcastle upon Tyne Hospitals NHS Foundation Trust, Newcastle upon Tyne, UK; 2 Department of Breast and Endocrine Surgery, University Hospital of South Manchester, Manchester, UK; 3 University Hospital of Wales, Cardiff, UK

## Abstract

This is the official guideline endorsed by the specialty associations involved in the care of head and neck cancer patients in the UK. This paper provides recommendations on the management of thyroid cancer in adults and is based on the 2014 British Thyroid Association guidelines.

**Recommendations:**

• Ultrasound scanning (USS) of the nodule or goitre is a crucial investigation in guiding the need for fine needle aspiration cytology (FNAC). (R)

• FNAC should be considered for all nodules with suspicious ultrasound features (U3–U5). If a nodule is smaller than 10 mm in diameter, USS guided FNAC is not recommended unless clinically suspicious lymph nodes on USS are also present. (R)

• Cytological analysis and categorisation should be reported according to the current British Thyroid Association Guidance. (R)

• Ultrasound scanning assessment of cervical nodes should be done in FNAC-proven cancer. (R)

• Magnetic resonance imaging (MRI) or computed tomography (CT) should be done in suspected cases of retrosternal extension, fixed tumours (local invasion with or without vocal cord paralysis) or when haemoptysis is reported. When CT with contrast is used pre-operatively, there should be a two-month delay between the use of iodinated contrast media and subsequent radioactive iodine (I^131^) therapy. (R)

• Fluoro-deoxy-glucose positron emission tomography imaging is not recommended for routine evaluation. (G)

• In patients with thyroid cancer, assessment of extrathyroidal extension and lymph node disease in the central and lateral neck compartments should be undertaken pre-operatively by USS and cross-sectional imaging (CT or MRI) if indicated. (R)

• For patients with Thy 3f or Thy 4 FNAC a diagnostic hemithyroidectomy is recommended. (R)

• Total thyroidectomy is recommended for patients with tumours greater than 4 cm in diameter or tumours of any size in association with any of the following characteristics: multifocal disease, bilateral disease, extrathyroidal spread (pT3 and pT4a), familial disease and those with clinically or radiologically involved nodes and/or distant metastases. (R)

• Subtotal thyroidectomy should not be used in the management of thyroid cancer. (G)

• Central compartment neck dissection is not routinely recommended for patients with papillary thyroid cancer without clinical or radiological evidence of lymph node involvement, provided they meet all of the following criteria: classical type papillary thyroid cancer, patient less than 45 years old, unifocal tumour, less than 4 cm, no extrathyroidal extension on ultrasound. (R)

• Patients with metastases in the lateral compartment should undergo therapeutic lateral and central compartment neck dissection. (R)

• Patients with follicular cancer with greater than 4 cm tumours should be treated with total thyroidectomy. (R)

• I^131^ ablation should be carried out only in centres with appropriate facilities. (R)

• Serum thyroglobulin (Tg) should be checked in all post-operative patients with differentiated thyroid cancer (DTC), but not sooner than six weeks after surgery. (R)

• Patients who have undergone total or near total thyroidectomy should be started on levothyroxine 2 µg per kg or liothyronine 20 mcg tds after surgery. (R)

• The majority of patients with a tumour more than 1 cm in diameter, who have undergone total or near-total thyroidectomy, should have I^131^ ablation. (R)

• A post-ablation scan should be performed 3–10 days after I^131^ ablation. (R)

• Post-therapy dynamic risk stratification at 9–12 months is used to guide further management. (G)

• Potentially resectable recurrent or persistent disease should be managed with surgery whenever possible. (R)

• Distant metastases and sites not amenable to surgery which are iodine avid should be treated with I^131^ therapy. (R)

• Long-term follow-up for patients with differentiated thyroid cancer (DTC) is recommended. (G)

• Follow-up should be based on clinical examination, serum Tg and thyroid-stimulating hormone assessments. (R)

• Patients with suspected medullary thyroid cancer (MTC) should be investigated with calcitonin and carcino-embryonic antigen levels (CEA), 24 hour catecholamine and nor metanephrine urine estimation (or plasma free nor metanephrine estimation), serum calcium and parathyroid hormone. (R)

• Relevant imaging studies are advisable to guide the extent of surgery. (R)

• RET (Proto-oncogene tyrosine-protein kinase receptor) proto-oncogene analysis should be performed after surgery. (R)

• All patients with known or suspected MTC should have serum calcitonin and biochemical screening for phaeochromocytoma pre-operatively. (R)

• All patients with proven MTC greater than 5 mm should undergo total thyroidectomy and central compartment neck dissection. (R)

• Patients with MTC with lateral nodal involvement should undergo selective neck dissection (IIa–Vb). (R)

• Patients with MTC with central node metastases should undergo ipsilateral prophylactic lateral node dissection. (R)

• Prophylactic thyroidectomy should be offered to RET-positive family members. (R)

• All patients with proven MTC should have genetic screening. (R)

• Radiotherapy may be useful in controlling local symptoms in patients with inoperable disease. (R)

• Chemotherapy with tyrosine kinase inhibitors may help in controlling local symptoms. (R)

• For individuals with anaplastic thyroid carcinoma, initial assessment should focus on identifying the small proportion of patients with localised disease and good performance status, which may benefit from surgical resection and other adjuvant therapies. (G)

• The surgical intent should be gross tumour resection and not merely an attempt at debulking. (G)

## Differentiated thyroid cancer

### Introduction

Thyroid nodules are common, the incidence of palpable nodules in women and men being approximately 5 and 1 per cent, respectively. Use of ultrasound scanning (USS) substantially increases their detection in the general population to approximately 50–70 per cent. Thyroid cancer remains rare, with an incidence in the UK of approximately 5 per 100 000 women and 2 per 100 000 men. Thyroid cancer is the most common endocrine malignancy, but accounts for only 1 per cent of all malignancies. Evidence suggests an increasing incidence; however, the survival rates remain static.

Long-term prognosis for differentiated thyroid cancer (DTC) is excellent, with survival rates for adults being 92–98 per cent at 10-year follow-up. However, 5–20 per cent of patients develop local or regional recurrence requiring further treatment and 10–15 per cent go on to develop distant metastases. Factors influencing prognosis include gender, age at presentation, histology and tumour stage. Accurate diagnosis, treatment and long-term follow-up are essential to achieve and maintain excellent survival rates.

There have been several sets of detailed guidelines published on the diagnosis and management of thyroid cancer. Two key ones are the Guidelines for the Management of Thyroid Cancer (2014) by the British Thyroid Association and Royal College of Physicians,^1^ and the Revised American Thyroid Association Guidelines (2016).^2^ These documents are extensive and cover every aspect of care in great detail. Given differences in presentation, pathophysiology and outcomes, separate guidelines exist for children with DTC,^3^ and consensus statements on the various surgical interventions.^4^ Patients may initially be seen by a surgeon, endocrinologist, clinical oncologist or nuclear medicine physician, who must be a core member of the thyroid cancer multidisciplinary team (MDT). The goals of treatment for DTC are set out in Box I.

BOX I **GOALS OF TREATMENT FOR DTC**

• Remove the primary tumour and involved lymph nodes
• Minimise treatment related morbidity
• Allow accurate staging of the disease
• Facilitate post-operative treatment with radioactive iodine in appropriate patients
• Enable long-term surveillance for disease recurrence
• Minimise the risk of disease recurrence and distant metastases

### Clinical presentation

In all cases, a detailed history is required. Clinical features associated with an increased risk of malignancy in individuals with a thyroid nodule include:

• age younger than 20 or older than 60 years
• firmness of the nodule on palpation
• rapid growth
• fixation to adjacent structures
• vocal cord paralysis
• associated lymphadenopathy
• history of neck irradiation
• family history of thyroid cancer
• history of Hashimoto's thyroiditis (risk factor for thyroid lymphoma).

#### Symptoms warranting immediate referral

Patients presenting with airway compromise, including stridor, associated with a thyroid nodule or goitre should be referred for an immediate opinion.

#### Symptoms warranting urgent general practitioner (GP) referral (two-week wait rule)

Patients presenting with hoarseness of voice or a change in their voice associated with a thyroid nodule or goitre, children with a thyroid nodule, individuals with cervical lymphadenopathy associated with a thyroid nodule or a painless thyroid mass, which is rapidly enlarging over a period of weeks should be referred for an urgent opinion.

### Investigation

#### Recommended clinical investigations

These include:

• Clinical evaluation of thyroid, cervical and supraclavicular nodes
• Thyroid-stimulating hormone (TSH)
• Ultrasound of the nodule (Table I)
• Fine needle aspiration cytology (FNAC) if ultrasound features are suspicious of malignancy
• Documented cytological score (Table II). A core biopsy (with or without USS guidance) is warranted if a diagnosis of lymphoma is suspected
• Calcitonin only in suspected cases of medullary thyroid cancer (MTC) (routine use not recommended)
• Pre-operative vocal cord check
• Note that a serum thyroglobulin (Tg) is not recommended.

**Table I:** U grading of thyroid nodules

U1 normal	U2 benign	U3 indeterminate/equivocal	U4 suspicious	U5 malignant
Normal thyroid tissue	Halo Iso-echoic or mildly hyper-echoic Cystic change ± ring down sign Micro-cystic/spongiform Peripheral egg shell calcification Peripheral vascularity	Homogeneous Hyper-echoic Solid, halo (follicular lesion) Equivocal echogenic foci Cystic change mixed/central vascularity	Solid Hypo-echoic or very hypo-echoic Disrupted peripheral calcification Lobulated outline	Solid Hypo-echoic Lobulated or irregular outline Micro-calcification Globular calcification Intra-nodular vascularity Shape (taller >wide) Characteristic associated lymphadenopathy
No follow-up required	No follow-up required – routine FNAC not recommended, unless high level of clinical suspicion of thyroid cancer	FNAC	FNAC	FNAC

FNAC = fine needle aspiration cytology

**Table II:** Thyroid FNAC diagnostic categories

Thy 1	Thy 2	Thy 3		Thy 4	Thy 5
		Thy 3F	Thy 3A		
Non-diagnostic	Non-neoplastic, e.g. colloid nodule or thyroiditis	Follicular lesion	Atypia present	Suspicious of thyroid cancer	Diagnostic of thyroid cancer
Repeat FNAC	No follow-up if no suspicious US features and no clinical suspicion of thyroid cancer	Diagnostic hemithyroidectomy* Consider total thyroidectomy in lesions >4 cm where incidence of malignancy is higher	Repeat ultrasound and FNAC If second Thy 3A cytology obtained, discuss at MDT and consider diagnostic hemithyroidectomy*	Discuss at MDT Diagnostic hemithyroidectomy*	Discuss at MDT Appropriate further investigations for staging where indicated Total thyroidectomy ± central node clearance in appropriate high risk patients

* Hemithyroidectomy consists of removal of a thyroid lobe and the isthmus

#### Ultrasound of thyroid nodules

Ultrasound is very useful in the investigation of thyroid nodules and should be used to guide the need for further investigation including FNAC. Ultrasound-guided FNAC increases the yield of diagnostic cytology significantly. Current guidelines recommend that ultrasonographers use the U grade (Table I) to classify nodules according to ultrasound appearances.^5^

#### Ultrasound evaluation of cervical lymphadenopathy

Pathological studies suggest that microscopic lymph node metastases are very common in papillary thyroid cancer (PTC). However, macroscopic disease is less common (20–50 per cent). Pre-operative ultrasonography is effective in identifying suspicious nodes in approximately 20–30 per cent of patients with PTC and may alter the surgical approach. FNAC of suspicious nodes is recommended. Tg estimation of cystic fluid may be of use in the absence of sufficient diagnostic material.

**Recommendations**

• Ultrasound scanning of the nodule or goitre is a crucial investigation in guiding the need for FNAC (R)
• FNAC should be considered for all nodules with suspicious ultrasound features (U3–U5). If a nodule is smaller than 10 mm in diameter, USS-guided FNAC is not recommended unless clinically suspicious lymph nodes on USS are also present (R)
• Cytological analysis and categorisation should be reported according to the current British Thyroid Association Guidance (R)
• Ultrasound scanning assessment of cervical nodes should be done in FNAC-proven cancer (R)
• Magnetic resonance imaging (MRI) or computed tomography (CT) should be done in suspected cases of retrosternal extension, fixed tumours (local invasion with or without vocal cord paralysis) or when haemoptysis is reported. When CT with contrast is used pre-operatively, there should be a two-month delay between the use of iodinated contrast media and subsequent radioactive iodine therapy (R)
• Fluoro-deoxy-glucose-positron emission tomography imaging is not recommended for routine evaluation (G)

### Staging

The tumour, nodes and metastases (TNM) staging system (Table III) is used to stage thyroid cancers and this should be used in all cases. Post-operatively, an ‘R’ classification can be given which indicates the amount of residual disease present. The TNM classification can then be used in combination with patient characteristics to define likely prognosis (Table IV).

**Table III:** Tumour, nodes and metastases 7th edition staging system for differentiated thyroid cancer

T stage – primary tumour	TX primary tumour cannot be assessed T0 no evidence of primary tumour T1 tumour ≤2 cm in greatest dimension limited to the thyroid T1a tumour ≤1 cm, limited to the thyroid T1b tumour >1 cm but ≤2 cm in greatest dimension, limited to the thyroid T2 tumour >2 cm but ≤4 cm in greatest dimension, limited to the thyroid T3 tumour >4 cm in greatest dimension limited to the thyroid or any tumour with minimal extrathyroidal extension (e.g. extension to sternothyroid muscle or peri-thyroid soft tissues) T4 tumour of any size extending beyond the thyroid capsule T4a tumour invades subcutaneous soft tissues, larynx, trachea, oesophagus or recurrent laryngeal nerve T4b tumour invades pre-vertebral fascia or encases carotid artery or mediastinal vessel
N stage – regional lymph nodes (cervical or upper mediastinal)	NX regional lymph nodes cannot be assessed N0 no regional lymph node metastasis N1 regional lymph node metastasis N1a metastases to level VI (pretracheal, paratracheal and prelaryngeal/Delphian lymph nodes) N1b metastases to unilateral, bilateral, or contralateral cervical (levels I–IV or V) or retropharyngeal or superior mediastinal lymph nodes (level VII)
M stage – distant metastases	MX distant metastases cannot be assessed M0 no distant metastasis M1 distant metastasis
R stage – residual disease	RX cannot assess presence of residual primary tumour R0 no residual primary tumour R1 microscopic residual primary tumour R2 macroscopic residual primary tumour

MDT = multidisciplinary team

**Table IV:** Group staging and survival for differentiated thyroid cancer

Stage	Age <45 years	Age >45 years	10-year survival (%)
I	Any T, any N, M0	T1, N0, M0	98.5
II	Any T, any N, M1	T2, N0, M0	98.8
III		T3, N0, M0 or T1–3, N1a, M0	99.0
*IVA		T4a, any N, M0 or T1–3, N1b, M0	75.9
IVB		T4b, any N, M0	62.5
IVC		Any T, any N, M1	63.0

* Undifferentiated or anaplastic carcinomas are all stage IV

### Surgery

Surgeons performing operations for confirmed or suspected thyroid cancer should be core members of the thyroid cancer MDT and should perform a minimum of 20 thyroidectomies per year. Complex surgery and lymph node surgery should be undertaken by nominated surgeons in the cancer centre with specific training in, and experience of, thyroid oncology. All patients with suspected or confirmed thyroid cancer should have pre-operative imaging with ultrasound. Cross-sectional imaging with CT or MRI may also be indicated.

In the context of thyroid cancer, surgery may be diagnostic (e.g. hemithyroidectomy following Thy 3 or Thy 4 cytology) or therapeutic.

#### Thyroid surgery for papillary thyroid cancer (PTC)

A strategy for the surgical treatment of PTC is detailed in Table V. All cases should be discussed pre-operatively at the thyroid cancer MDT.

**Table V:** Initial surgery for papillary thyroid carcinoma

	Tumour <4 cm	Tumours >/=4 cm	T3 and T4 tumours +N1 level VI nodes, M1
Recommendation	With no other clinical features such as age >45 years, extrathyroidal spread, nodal involvement, angioinvasion, multifocality, distant metastases	Papillary cancer diagnosed following hemithyroidectomy, multifocal disease, thyroid radiation in childhood, familial disease (first degree relative)	Treat all above tumours as high risk
Hemithyroidectomy	Yes	No	No
Total thyroidectomy	Discuss at MDT	Completion total thyroidectomy	Yes
Prophylactic level VI nodal dissection	No	Personalised decision making	Yes
Therapeutic level VI nodal dissection (clinically involved)	Yes	Yes	Yes

#### Initial surgery for follicular thyroid cancer

The majority of patients undergoing surgery for follicular thyroid cancer will be undiagnosed at the time of the initial surgery (Thy 3). Frozen section histology cannot currently reliably differentiate benign follicular lesions from follicular thyroid cancer, and therefore this strategy is not recommended. An operative strategy for surgical treatment of follicular cancer is outlined in Table VI.

**Table VI:** Initial surgery for follicular thyroid cancer

	Clinical details	
Recommendation	Low-risk patient (with all of following) <45 years >1–≤4 cm Minimally invasive No angioinvasion No extracapsular invasion No extrathyroidal spread	High-risk patient (one or more of the following) >45 years Tumour >4 cm Extra-capsular invasion Extrathyroidal disease Widely invasive Angioinvasion Hurthle cell tumours
Hemithyroidectomy	Yes	No
Total thyroidectomy	No	Yes
Level VI nodal dissection	No	Only where clinically involved nodes present

Low-risk patients with a diagnosis of minimally invasive tumour less than 4 cm following hemithyroidectomy do not require further treatment. Hurthle cell cancers (follicular oncocytic) tend to be more aggressive and should be treated by total (completion) thyroidectomy (see Table VI).

#### Management of lymph nodes in differentiated thyroid cancer (DTC)

Prophylactic level VI lymph node dissection is associated with a higher incidence of recurrent laryngeal nerve damage and long-term permanent hypoparathyroidism.^6^ It is therefore not routinely recommended, but in individuals with high-risk tumours, this should be discussed in the spirit of personalised decision making. Prophylactic level VI nodal dissection is not recommended in low risk, small papillary and most follicular cancers.

Prophylactic level VI nodal dissection is recommended in patients with known involved lateral nodes. Therapeutic level VI nodal dissection is recommended when the presence of lymph node metastasis is confirmed.

Clinically involved lateral cervical lymph nodes should be managed by selective neck dissection (levels II–V). Involvement of level I or  VII node is rare in DTC and should only be dissected if involved. Prophylactic lateral neck compartment dissection for node negative patients is not recommended.

#### Completion thyroidectomy

Completion thyroidectomy is not needed in low-risk, unifocal, intrathyroidal tumours less than 4 cm in diameter, with clinically negative lymph nodes.

#### Locally advanced disease

Where possible, locally advanced disease should be resected. Preservation of recurrent laryngeal nerves should be attempted in almost all cases. Extensive resection of trachea, larynx and oesophagus should be considered if potentially curative. Where disease is unresectable, radiotherapy and radioiodine should be considered.

#### Microcarcinomas

**Fig. 1:**
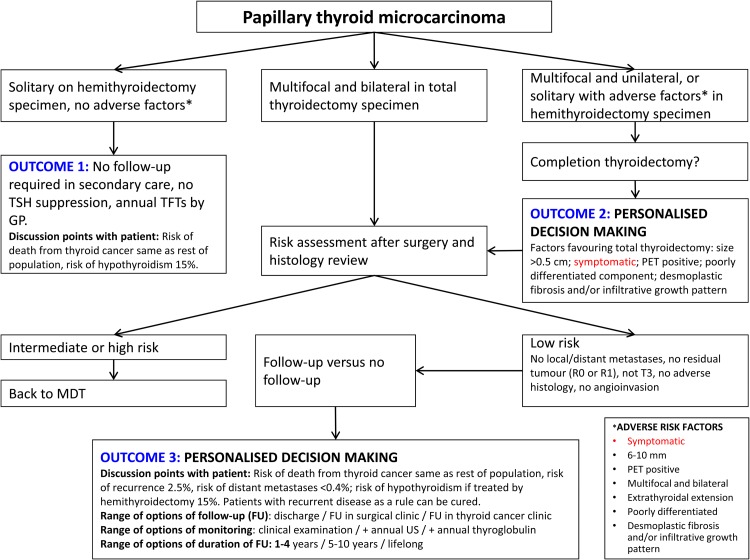
Flow diagram outlining management of papillary microcarcinomas. Multiple risk factors may tip the balance in favour of total thyroidectomy.

Microcarcinomas are differentiated thyroid carcinomas less than 10 mm in maximum dimension and are predominantly papillary carcinomas. The management of papillary microcarcinomas is outlined in Figure 1.

**Recommendations**

• In patients with thyroid cancer, assessment of extrathyroidal extension and lymph node disease in the central and lateral neck compartments should be undertaken pre-operatively by USS and cross-sectional imaging (CT or MRI) if indicated (R)
• For patients with Thy 3f or Thy 4 FNAC a diagnostic hemithyroidectomy is recommended (R)
• Total thyroidectomy is recommended for patients with tumours greater than 4 cm in diameter, or tumours of any size in association with any of the following characteristics: multifocal disease, bilateral disease, extrathyroidal spread (pT3 and pT4a), familial disease, and those with clinically or radiologically involved nodes and/or distant metastases (R)
• Subtotal thyroidectomy should not be used in the management of thyroid cancer (G)
• Central compartment neck dissection is not recommended for patients without clinical or radiological evidence of lymph node involvement, provided they meet all of the following criteria: classical type PTC, below 45 years, unifocal tumour, less than 4 cm, no extrathyroidal extension on US (R)
• Patients with metastases in the lateral compartment should undergo therapeutic lateral and central compartment neck dissection (R)
• Patients with follicular tumours greater than 4 cm should be treated with total thyroidectomy (R)

### Post-operative management

After total or near total thyroidectomy patients should be commenced on suppressive doses of levothyroxine (2 µg/kg) or liothyronine 20 mcg tds in accordance with local protocols.

Calcium levels should be routinely checked within 24 hours and hypocalcaemia treated appropriately.

Thyroglobulin levels should be checked no earlier than six weeks after surgery.

All patients with thyroid cancer should be clinically staged using the TNM classification and also scored using one of the clinicopathological scoring systems to enable planned follow-up, identification of high risk patients and those who would benefit from radio-iodine therapy. In addition, all patients should have access to a thyroid cancer clinical nurse specialist and be given written information.

Persistent voice dysfunction should be investigated and referral to a specialised practitioner for assessment and speech therapy sought.

Patients with long-term hypocalcaemia (hypoparathyroidism) should have their calcium levels regularly monitored either in association with an endocrinologist or with their GP.

Following surgery, initial post-operative risk stratification for risk of recurrence can occur.

Low-risk patients have the following characteristics:

• No local or distant metastases
• All macroscopic tumours have been resected, i.e. R0 or R1 resection
• No tumour invasion of locoregional tissues or structures
• The tumour does not have aggressive histology (tall cell or columnar cell PTC, diffuse sclerosing PTC, poorly differentiated elements) or angioinvasion.

Intermediate-risk patients have any of the following characteristics:

• Microscopic invasion of tumour into the peri-thyroidal soft tissues (T3) at initial surgery
• Cervical lymph node metastases (N1a or N1b)
• Tumour with aggressive histology (tall cell or columnar cell PTC, diffuse sclerosing PTC, poorly differentiated elements) or angioinvasion.

High-risk patients have any of the following characteristics:

• Extrathyroidal invasion
• Incomplete macroscopic tumour resection (R2)
• Distant metastases. (M1)

### Radioiodine (I^131^) ablation and external beam radiotherapy (EBR) in DTC

The current recommendations with regards to I^131^ ablation following total thyroidectomy are outlined in Table VII.

**Table VII:** Indications for I^131^ ablation following total thyroidectomy for differentiated thyroid cancer

Recommendation	Clinical details
Definite I^131^ ablation	Tumour >4 cm Any tumour size with gross extrathyroidal extension Distant metastases present
Probable I^131^ ablation Consider on individual case merit (MDT)	Risk factors indicating higher risk of recurrence where I^131^ should be considered include: Large tumour size Extrathyroidal extension Unfavourable cell type (tall cell, columnar or diffuse sclerosing papillary cancer, poorly differentiated elements) Widely invasive histology Multiple lymph node involvement, large size of involved lymph nodes, high ratio of positive-to-negative nodes, extracapsular nodal involvement
No I^131^ ablation (all criteria must be met)	Tumour <1 cm unifocal or multifocal Histology classical papillary or follicular variant of papillary carcinoma, or follicular carcinoma Minimally invasive without angioinvasion No invasion of thyroid capsule (extrathyroidal extension)

Patients should be prepared for I^131^ by having a low-iodine diet for one to two weeks prior to treatment. Recombinant TSH (rhTSH) therapy prior to I^131^ is preferable to thyroid hormone withdrawal, and is preferred by patients, providing they meet the following criteria: pT1 to T3, pN0 or NX or N1, and M0 and R0 (no microscopic residual disease). Pregnancy should be excluded prior to giving I^131^. A post-ablation scan should be performed after I^131^ when residual activity levels permit satisfactory imaging. Practically, this is generally 2–10 days following treatment.

Following I^131^, TSH should be suppressed to <0.1 mIU/l pending dynamic risk stratification at 9–12 months.

Adjuvant EBR should be considered in unresectable tumours in addition to I^131^ and where there is residual disease following surgical resection even if the residual tumour concentrates I^131^.

In the 9 to 12 months following surgery and I^131^ for DTC with an R0 resection, patients should undergo dynamic risk stratification (Table VIII). Patients are then categorised as having either an excellent response, an indeterminate response or an incomplete response.

**Table VIII:** Dynamic risk stratification following treatment for DTC and TSH suppression targets for patients treated with total thyroidectomy and I^131^ ablation with R0 resection

Excellent response	Indeterminate response	Incomplete response
All the following: Suppressed and stimulated Tg < 1 lg/l* Neck US without evidence of disease Cross-sectional and/or nuclear medicine imaging negative (if performed)	Any of the following: Suppressed Tg < 1 lg/l* and stimulated Tg ≥ 1 and <10 lg/l* Neck US with non-specific changes or stable sub centimetre lymph nodes Cross-sectional and/or nuclear medicine imaging with non-specific changes, although not completely normal	Any of the following: Suppressed Tg ≥ 1 lg/l* or stimulated Tg ≥ 10 lg/l* Rising Tg values Persistent or newly identified disease on cross-sectional and/or nuclear medicine imaging
Low risk Maintain TSH 0.3–2.0 mIU/l	Intermediate risk Suppress TSH 0.1–0.5 mIU/l for 5–10 years then reassess	High risk Suppress TSH < 0.1 mIU/l indefinitely

* Assumes the absence of interference in the Tg assay. Tg = thyroglobulin; TSH = thyroid stimulating hormone; US = ultrasound

#### Monitoring Tg levels

Thyroglobulin monitoring is most effective following total or near total thyroidectomy and I^131^ and is an important modality in detecting residual or recurrent disease. Physicians should be aware that Tg estimations vary according to the assay method, the individual laboratory and the presence of anti-Tg antibodies and take these considerations into account when evaluating Tg levels in individual patients.

The patient should have their Tg levels checked at 6–12 monthly intervals. Rising Tg levels are highly suspicious of recurrent disease. Thyroglobulin evaluation is most effective following TSH stimulation, either by direct rhTSH stimulation or by withdrawal of thyroid hormone replacement.

Following total or near total thyroidectomy and I^131^ ablation, low-risk patients with undetectable Tg levels on TSH suppression should have a TSH-stimulated Tg assessment along with ultrasound of cervical nodes at 9–12 months following I^131^ ablation. If Tg levels remain undetectable following TSH stimulation, then future recurrent disease is highly unlikely and patients may revert to yearly Tg estimation whilst remaining on TSH suppression.

A rise in Tg may be suggestive of recurrent or residual disease, but is usually from a thyroid remnant. In low-risk patients, an expectant policy can be maintained and repeated TSH stimulated assessment performed, with the expectation that Tg levels will fall. Rising or persistently elevated Tg needs further evaluation.

The use of rhTSH-stimulated Tg estimation or rhTSH I^131^ therapy is necessary in the following cases: hypopituitarism, functional metastases (suppressing TSH), severe angina, advanced disease (frail patient) and history of psychiatric disturbance from hypothyroidism.

**Recommendations**

• I^131^ ablation or therapy should be carried out only in centres with appropriate facilities (R)
• Serum Tg should be checked in all post-operative patients with DTC, but not earlier than six weeks after surgery (R)
• Patients who have undergone total or near total thyroidectomy should be started on levothyroxine 2 µg/kg or liothyronine 20 mcg tds after surgery (R)
• The majority of patients with a tumour more than 1 cm in diameter, who have undergone total or near-total thyroidectomy, should have I^131^ ablation or therapy (R)
• A post-ablation scan should be performed 3–10 days after I^131^ ablation (R)
• Post-therapy dynamic risk stratification at 9–12 months is used to guide further management (G)

### Persistent and recurrent disease, locoregional recurrence and distant metastases

Potentially resectable disease is best managed by surgery (including local cervical nodes and soft tissue disease in the neck), followed by I^131^. Residual disease not amenable to resection or resistant to I^131^ therapy is best treated with high dose palliative EBR.

Therapeutic central compartment, with or without lateral compartment, nodal clearance should therefore be performed for all persistent or recurrent disease confined to the neck. Impalpable nodes greater than 5–8 mm seen on USS or cross-sectional imaging following I^131^ therapy should be considered for removal. Removing nodes less than 5–8 mm has not be shown to be of benefit.

Where technically feasible, tumours invading the aero-digestive tract should be resected in combination with radiotherapy. Outcome is very dependent on completeness of resection and preservation of function. Great care should therefore be taken in the selection and discussion of such patients at the MDT.

Distant metastases develop in 5–23 per cent of patients with DTC. Sites not amenable to surgical resection should be treated with I^131^ therapy. Long-term survival may be expected in patients whose tumours take up I^131^. Distant metastases are usually seen in the lungs and bones. There is no maximum limit to the cumulative dose of I^131^ that patients with persistent disease may receive and pulmonary fibrosis appears to be a rare side effect. Surgical resection of bony metastases should be considered (especially in patients below 45 years of age). Metastases not cured by I^131^ should be treated with EBR. Other modalities such as intra-arterial embolisation, pamidronate infusion, radiofrequency ablation or vertebroplasty may be considered in cases of painful lesions.

**Recommendations**

• Potentially resectable recurrent or persistent disease should be managed with surgery whenever possible (R)
• Distant metastases and sites not amenable to surgery, which are iodine avid should be treated with I^131^ therapy (R)

### Long-term follow-up

Lifelong follow-up of DTC is recommended to monitor for late recurrence (often treatable and curable), effects of long-term TSH suppression (atrial fibrillation and osteoporosis) and late side effects of I^131^. Clinical examination and history, Tg determination, TSH suppression and where necessary calcium monitoring should all be performed. Ultrasound scanning as per established protocols may also be undertaken.

**Recommendations**

• Long-term follow-up for patients with DTC is recommended (G)
• Follow-up should be based on clinical examination, serum Tg and TSH assessments (R)

## Medullary thyroid cancer

### Introduction

Medullary thyroid cancer (MTC) is a rare cancer (approximately 1–3 per cent of all thyroid cancer cases). All cases should be referred for surgical treatment to the designated cancer centre of the Thyroid Cancer Network. Twenty-five per cent of MTC cases are familial (MEN2A, MEN2B and familial non-MEN MTC). Genetic screening (*RET* mutation testing) of all patients is mandatory and the assessment, investigation and treatment of family members at potential risk requires a multidisciplinary approach within the cancer centre.^7^

### Clinical presentation

Patients usually present clinically with a thyroid nodule or neck mass with or without cervical lymphadenopathy (in the same fashion as with DTC). History however, may reveal other symptoms such as flushing, loose stools or diarrhoea (which suggest MTC) and is vitally important in determining a potential familial element. FNAC may be diagnostic (when combined with calcitonin staining in suspicious cases), but often is reported as Thy 3.

### Investigation

When MTC is suspected (or proven) patients must undergo the following investigations prior to surgery^8^:

• Calcitonin and CEA levels
• Twenty-four-hours urine estimation of catecholamines and nor metanephrines (or plasma nor metanephrines) to identify or exclude phaeochromocytoma
• Serum calcium and parathyroid hormone (PTH) to identify or exclude hyperparathyroidism
• CT, MRI or USS of the neck are indicated as they may help guide the extent of surgical resection at initial surgery
• RET proto-oncogene mutational analysis should be performed after surgery once diagnosis is established, even in the absence of a familial history.

### Staging

TNM staging for MTC follows the same criteria as for DTC (Table IX).

**Recommendations**

• Patients with suspected MTC should be investigated with calcitonin and CEA levels, 24 hours catecholamine and nor metanephrine urine estimation (or plasma free nor metanephrine estimation), serum calcium and PTH (R)
• Relevant imaging studies are advisable to guide the extent of surgery (R)
• RET proto-oncogene analysis should be performed after surgery (R)

**Table IX:** Group staging for medullary thyroid cancer

Stage I	T1, N0, M0
Stage II	T2, T3, T4, N0, M0
Stage III	Any T, N1, M0
Stage IV	Any T, any N, M1

### Management-surgery for MTC

All patients with MTC should undergo^8^:

• Total thyroidectomy and central compartment node clearance (level VI). This should be performed even in the presence of disseminated metastases to control local disease.
• In the presence of central compartment lymph node metastases, ipsilateral prophylactic neck dissection is recommended as up to 70 per cent of patients will have lateral nodal metastases.
• Patients with clinically involved lateral compartment nodes should have a therapeutic lateral neck dissection to eradicate local disease.
• All T2–T4 tumours should also undergo prophylactic bilateral selective neck dissection IIa–Vb.
• Intra-thoracic disease below the level of the brachiocephalic vein should be resected via sternotomy where feasible.
• Prophylactic thyroidectomy should be offered to RET-positive family members. Timing and extent of surgery are dependent on genotype (codon mutation), the calcitonin level and age at detection of RET positivity.

### Persistent or recurrent MTC

Calcitonin levels are most informative six months after initial surgery. It is important to distinguish persistent locoregional disease (following either inadequate initial surgery or local lymph node metastases) from distant disease.

Early local recurrence following adequate local surgery (total thyroidectomy and level VI nodes) is unusual. The likely source of raised calcitonin in this circumstance is the lateral compartment cervical nodes, i.e. persistent disease. When indicated, re-operation including further central compartment surgery and lateral neck node dissection should be performed. The primary aim should always be to control local disease.

CT, MRI, USS, selective arteriography, I^131^-metaiodobenzylguanidine, ^18^Fluoro-deoxy-glucose positron emission tomography, In^111^-octreotide and direct laparoscopic visualisation of the liver may all be useful in identifying the source of a raised calcitonin, but their use in patients with calcitonin levels <400–500 pg/ml is unlikely to identify metastases. When indicated, isolated metastases should be considered for surgical resection.

**Recommendations**

• All patients with known or suspected MTC should have serum calcitonin and biochemical screening for phaeochromocytoma pre-operatively (R)
• All patients with proven MTC >5 mm should undergo total thyroidectomy and central compartment neck dissection (R)
• Patients with lateral nodal involvement should undergo selective neck dissection (IIa–Vb) (R)
• Patients with central node metastases should undergo ipsilateral prophylactic lateral node dissection (R)
• Prophylactic thyroidectomy should be offered to RET-positive family members (R)
• All patients with proven MTC should have genetic screening (R)

### Radiotherapy and chemotherapy

Radiotherapy is of use in controlling local symptoms in patients with inoperable disease and improving the relapse-free rate following central or lateral compartment surgery where residual disease is present macroscopically or microscopically.

Tyrosine kinase inhibitors can be effective in controlling symptoms in patients with metastatic disease.

Somatostatin analogues may be effective in alleviating the unpleasant gastrointestinal symptoms that patients with advanced cases of MTC experience.

**Recommendations**

• Radiotherapy may be useful in controlling local symptoms in patients with inoperable disease (R)
• Chemotherapy with tyrosine kinase inhibitors may help in controlling local symptoms (R)

### Follow-up

Lifelong follow-up is recommended for all patients with MTC. Screening should include calcitonin and CEA. Thyroid-stimulating hormone suppression is not necessary. Rising calcitonin levels should trigger investigations to identify potentially treatable metastatic disease.

## Anaplastic thyroid cancer

The prognosis of patients with anaplastic thyroid cancer (ATC) is poor. Many patients present with a history of a rapidly enlarging thyroid mass in a long-standing goitre. Diagnosis can be established by fine needle aspiration or core biopsy. Core biopsy will help differentiate ATC from thyroid lymphoma which can present in a similar manner.

Total thyroidectomy may be curative for very small cancers. In more advanced disease surgery may be of benefit if R0/R1 resection is achievable.^9^ External beam radiotherapy and chemotherapy may be used as adjuvant treatments in patients with R0/R1 resection and no evidence of distant disease. ‘Debulking’ surgery should be avoided when complete resection cannot be achieved. Palliative chemoradiation may be of some value in selected cases. Palliative care has a principal role in management of these patients.

**Recommendations**

• Initial assessment should focus on identifying the small proportion of patients with localised disease and good performance status, who may benefit from surgical resection and other adjuvant therapies (G)
• The surgical intent should be gross tumour resection and not merely an attempt at debulking (G)
